# Versatile use of microliths as a technological advantage in the miniaturization of Late Pleistocene toolkits: The case study of Neve David, Israel

**DOI:** 10.1371/journal.pone.0233340

**Published:** 2020-06-03

**Authors:** Iris Groman-Yaroslavski, Hong Chen, Cheng Liu, Ron Shimelmitz, Reuven Yeshurun, Jiying Liu, Xia Yang, Dani Nadel

**Affiliations:** 1 Zinman Institute of Archaeology, University of Haifa, Mount Carmel, Haifa, Israel; 2 Department of Cultural Heritage and Museology, Zhejiang University, Hangzhou, China; 3 Institute of Cultural Heritage and Museology, Zhejiang University, Hangzhou, China; Max Planck Institute for the Science of Human History, GERMANY

## Abstract

The miniaturization of stone tools, as reflected through the systematic production of bladelets and bladelet tools (microliths), characterized many industries of the Late Pleistocene, with the Levantine Epipalaeolithic serving as a well-studied example. It is commonly held that microliths were used as modular inserts in composite projectiles, while their incorporation in other tools for different tasks is generally overlooked, the latter aspect being the main focus of this paper. We present here a more inclusive approach through a case study of the Geometric Kebaran (Middle Epipalaeolithic, ca. 18,500–15,000 cal BP) site of Neve David, Mount Carmel, Israel. Recent excavations at the site exposed a variety of features, and one well-preserved shallow pit provided a large lithic assemblage with ca. 90 microliths. We studied this assemblage using both the low- and high- magnification use-wear protocols, accompanied by a range of experiments. Our results show that a) the fragmentation rate is very high in this assemblage (ca. 90%), b) most of the microliths have identifiable use-wear, c) the microliths were commonly used as inserts in composite projectiles, d) many microliths were used for functions not related to weaponry and hunting, such as wood-working, weed harvesting and meat processing. These findings strongly support the suggestion that the small insets, regardless of their specific type (trapeze, rectangle, backed/retouched bladelet), were used in a wide variety of composite tools. We argue that such a versatile approach and flexibility in the use of microliths reflect a technological advantage where a minimal set of microlithic types, produced in large numbers, could provide the required elements for weapons, as well as for a variety of cutting, processing and harvesting tools needed for mundane tasks at a large Middle Epipalaeolithic camp.

## 1. Introduction

The miniaturization of stone tools, as reflected through the systematic production of bladelets and bladelet tools (microliths), characterized many industries of the Late Pleistocene, with its sprouts emerging already in the Middle Stone Age of Africa, the Middle Paleolithic of Eurasia [1–4] and also manifested in small flake production in various contexts of the Pleistocene [5,6] (and references therein). The exploitation of microliths intensified at the end of the Late Pleistocene [1] with the Levantine Epipaleolithic (ca. 24,000–11,600 cal BP) being one of the more familiar cases [7–10].

Microlithic technology has been ascribed with several adaptive advantages. Clarkson and colleagues [1] recently presented a summary of these advantages addressing aspects of transportability, raw-material exploitation, manufacture procedures, standardization, haftability, maintainability, and reliability. These can be grouped into two interrelated sets: one addressing the advantages gained in the process of manufacturing the small implements and the second addressing the advantages related to their hafting and use in composite tools. Within this technological organization, the effort invested in manufacturing the haft is considered greater than the effort invested in the inserts. While the haft is assumed to have been kept for a long time, the microlithic inserts are assumed to have been regularly replaced and discarded. The practice of hafting using relatively easy exchangeable inserts was argued to provide an advantage for mobile hunter gatherers, equipping them with a highly reliable technology [2,11]. These advantages were already of significance from the onset of the systematic manufacture of bladelets and bladelet tools, and have become a hallmark of the technological organization at the end of the Pleistocene in cases such as the Epipaleolithic of the Levant and North Africa, where microliths usually formed the majority of the tools.

In the Levantine Epipaleolithic microliths were suggested to have been hafted in a variety of methods, usually reconstructed as inserts within projectiles [12–17]. Studies focusing on the typical damage indicative of their use as projectiles, termed diagnostic impact fractures (DIFs), indicate the utilization of different microlith types as components of composite projectiles in various ways, and even the use of a specific type of microlith in various hafting modes [18–20]. In parallel, several use-wear studies identified different functions of microliths, demonstrating that microlith technology encompassed a wide range of composite tools that may have been used in various activities. In fact, studies focusing on DIFs of large samples from Levantine sites such as Neve David, el-Wad Terrace and Ohalo II, show the use of such implements in composite projectiles in low frequencies—less than 10% [21,22] (Table 3 and Table 7 respectively). The use of microliths for various activities is demonstrated through studies from several regions and periods [23–26]; in the Levantine context, Macdonald's study of microliths from Geometric Kebaran sites in Jordan (ca. 18,500–15,000 cal BP) provides the most systematic evidence for their various uses [18]. These tools were also used as cutting and scraping implements of a range of target materials [27,28]. Noteworthy is the new and common function of large microliths as inserts in cereal harvesting tools during the later Epipaleolithic Natufian [29,30].

While these results demonstrate significant insights regarding the complexity of microliths use, they are still at the fringes of the common reconstructions that conceive microliths as components of hunting gear, thus likely overlooking the wider and diverse functional significance of these inserts. The current state in the research of microliths demonstrates the need for an approach incorporating a wide perspective. In this paper we focus directly on this issue by addressing the versatile use of microliths, as a leading and important component in the general advantage of microliths, alongside manufacturing efficiency and ease of haftability.

As a case study, a use-wear analysis was applied to an assemblage of microliths deriving from a small pit exposed during the renewed excavation at the Epipaleolithic site of Neve David, Mount Carmel, Israel [31]. We argue that providing a clear case of the complex and diverse functions of microliths as reflected through their use as projectiles, alongside their use for activities that are not related to weapons, will demonstrate the multifaceted tool-use inherent within the technological organization of Epipaleolithic groups. Based on these results, we therefore suggest that the importance of microliths should be re-evaluated in terms of their adaptive advantages and social significance, beyond simple cost-benefit calculations regarding hunting gear [32].

### 1.1 The site of Neve David

**Fig 1:**
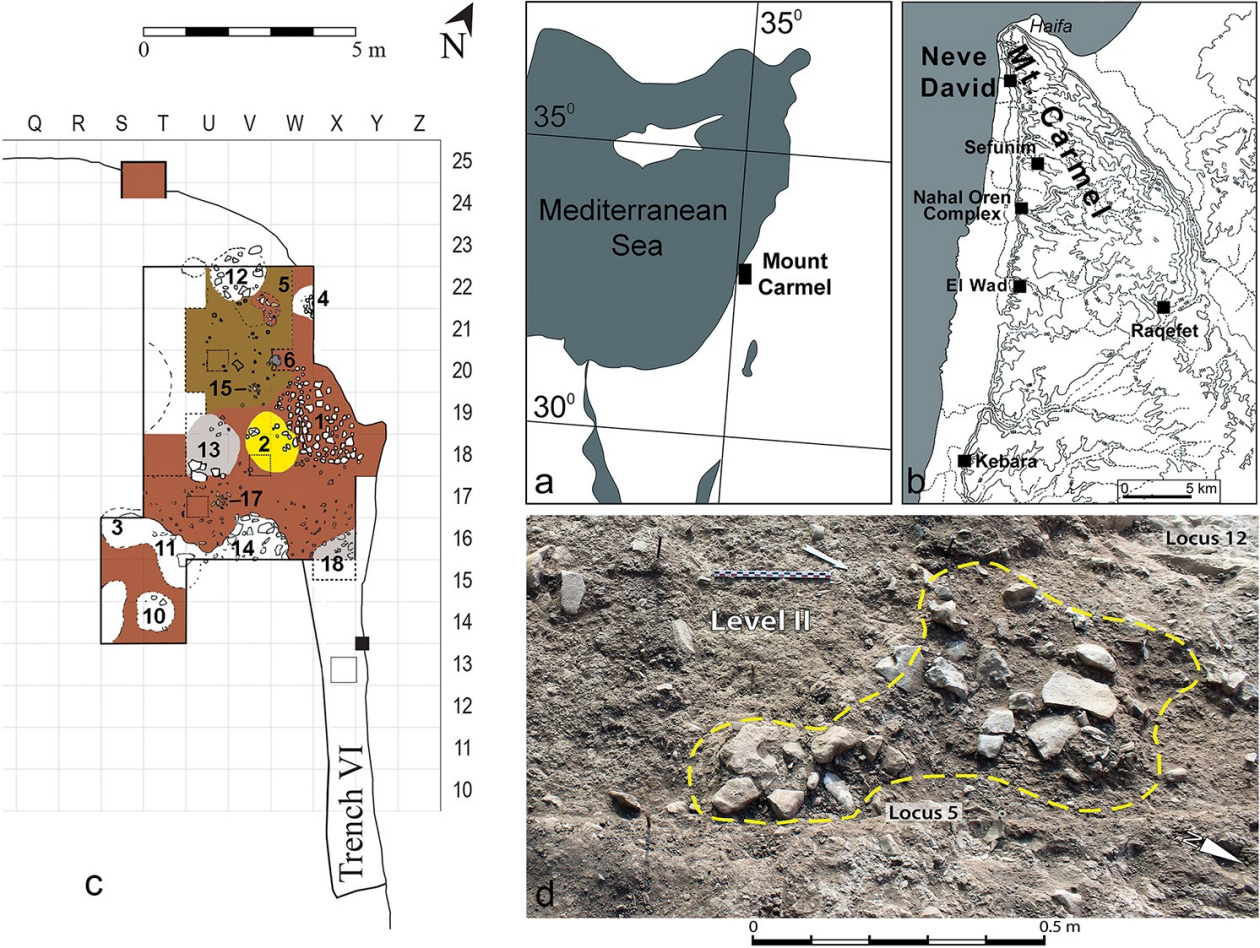
Location map of Neve David and Locus 5. (a) Location map of Mount Carmel. (b) Neve David and other contemporaneous sites mentioned in the text. (c) Excavation plan showing the location of Locus 5. (d) Field photo of Locus 5.

Neve David is a large open-air site assigned to the Geometric Kebaran (GK) phase of the Epipaleolithic period (ca. 18,500–15,000 BP). The site is situated at the foot of the western slope of Mount Carmel, on the north bank of Nahal Siah at its outlet to the coastal plain, in the modern city of Haifa, Israel (Fig 1). The site was first excavated over four seasons between 1984 and 1990 [33–36] and the renewed excavations commenced in 2014 [31].

The site is partially damaged by a modern road but it appears to have covered about 1,500 m^2^, and the thickness of the Epipaleolithic deposits seems to exceed 1.5 m. These are topped and disturbed by Holocene remains attributed to the Neolithic and later periods [37]. The Epipaleolithic horizon is characterized by a distinctive dark brown to reddish clay with high densities of lithics and faunal remains. The Epipaleolithic features include two human burials, one of which was associated with ground stone tools, a few arranged stones [33,38] and several small pits that are cut from and into the GK living levels; one of the pits, Locus 5, is the source of the lithic sample presented here. Samples retrieved from burnt animal bones yielded dates of 15,150–15,450 cal BP (Oxa-892) and 16,820–16,090 cal BP (Oxa-859) [34,38]; a renewed dating project is underway. A rich faunal assemblage, dominated by mountain gazelle (*Gazella gazella*) and Mesopotamian fallow deer (*Dama mesopotamica*) with low numbers of small game was found at the site [31,39].

Locus 5 is located in the northeast corner of the renewed central excavation area (Squares V-W/21-22; Fig 1C). Embedded in the GK layer, it is a pit with an irregular shape, about 1 x 0.5 m at the top and narrowing with depth. The feature is 15–18 cm deep. It was distinct from the surrounding GK sediments by a concentration of relatively large and complete lithic artifacts, small stones and relatively complete faunal finds (e.g., gazelle and fallow deer jaws, pelves and scapulae), all embedded in red-brown clay matrix (Fig 1D). Unlike the upper part of the locus which had no noticeable difference in matrix compared to the surrounding sediment (Level I), the lower parts were distinct from the surrounding matrix (belonging to Levels II and III) which was more yellow-brown and compact. Thus, the entire Locus 5 probably represents a pit-feature dug from Level I.

## 2. Materials and methods

### 2.1 The Locus 5 flint assemblage

No permits were required for the described study. The flint assemblage from Locus 5 comprises 840 pieces, excluding chunks and pieces smaller than 1 cm (Table 1) [40]. It is similar in composition to other studied flint samples at the site [35,40] by showing clear indication of on-site knapping with the presence of all debitage components; a high ratio of *ca*. 7 tools (N = 119) to cores (N = 18) is recorded. Cores and tools from Locus 5 were mainly produced of local flint sources (76.6%, N = 105), most probably from the Nahal Siah Shamir Formation outcrops, located within 100–700 m on the slopes above the site [41]. The density of lithic finds within the pit is high; by extrapolation it reaches ca. 300 cores/m^3^ and 1,980 tools/m^3^. The assemblage is marked by a high proportion of blades and bladelets, both in terms of blanks and of shaped tools, and more than half of the cores (66.7%, N = 12) exhibit blade/let scars.

**Table 1:** The flint assemblage from Locus 5.

Type	N	%
**Debitage**		
Cores	18	2.1%
Primary flakes	114	13.6%
Primary blades	18	2.1%
Primary bladelets	29	3.5%
Flakes	240	28.6%
Blades	41	4.9%
Bladelets	236	28.1%
Core trimming elements	24	2.9%
Burin spalls	1	0.1%
**Debitage total**	**721**	**85.8%**
**Tools**		
Non-microlithic tools	30	3.6%
Microliths	89	10.6%
**Tools total**	**119**	**14.2%**
**Total**	**840**	**100.0%**

The composition of the assemblage reflects a typical GK repertoire, as seen in several sites [10,20,42–45]. As in many Early and Middle Epipaleolithic sites, the fragmentation rate of microliths is very high (Table 2). In most sites cited here it is above 60%, and indeed such high rates represent the vulnerability of the delicate and fragile inserts. The rate may be affected by the function of the sites, post-depositional processes, as well as excavation methods (size of the sieves, dry/wet sieving, etc.). Even at the well-preserved and submerged microlithic-based site of Ohalo II, the rate is above 90% [46,47]. Clearly, any study of microliths from Epipaleolithic sites has to take into account the high fragmentation rate as an integral part of the archaeological record.

**Table 2:** Fragmentation rates of microliths from Neve David and selected geometric kebaran sites.

Site	Area	Fragmentation rate	References
Neve David Loc. 5	Mount Carmel	89.9% (n = 89)	[40]
Neve David sample	Mount Carmel	92.3% (n = 2714)	[40]
Ain Miri	Upper Galilee	74.7% (n = 1734)	[20]
Haon III	Central Jordan Valley	39.8% (n = 83)	[48]
Hayonim Terrace	Lower Galilee	60.8% (n = 423)	[49]
Hefziba	Northern Coastal Plain	71.4% (n = 9414)	[50]
*Ohalo II	Sea of Galilee	91.5% (n = 504)	(Nadel n.d.)

*that the earlier and well-preserved Ohalo II submerged site also has a very high fragmentation rate.

Regarding the Locus 5 microliths, the fragmentation rate of 89.9% is somewhat lower than the average for several studied samples at the site (92.3%) (Table 2) [40]; only nine microliths are complete, all geometrics. With all given limitations, such a fragmentation rate is common and should not be considered an obstacle in studying and interpreting the use-wear and function of the microliths.

**Fig 2:**
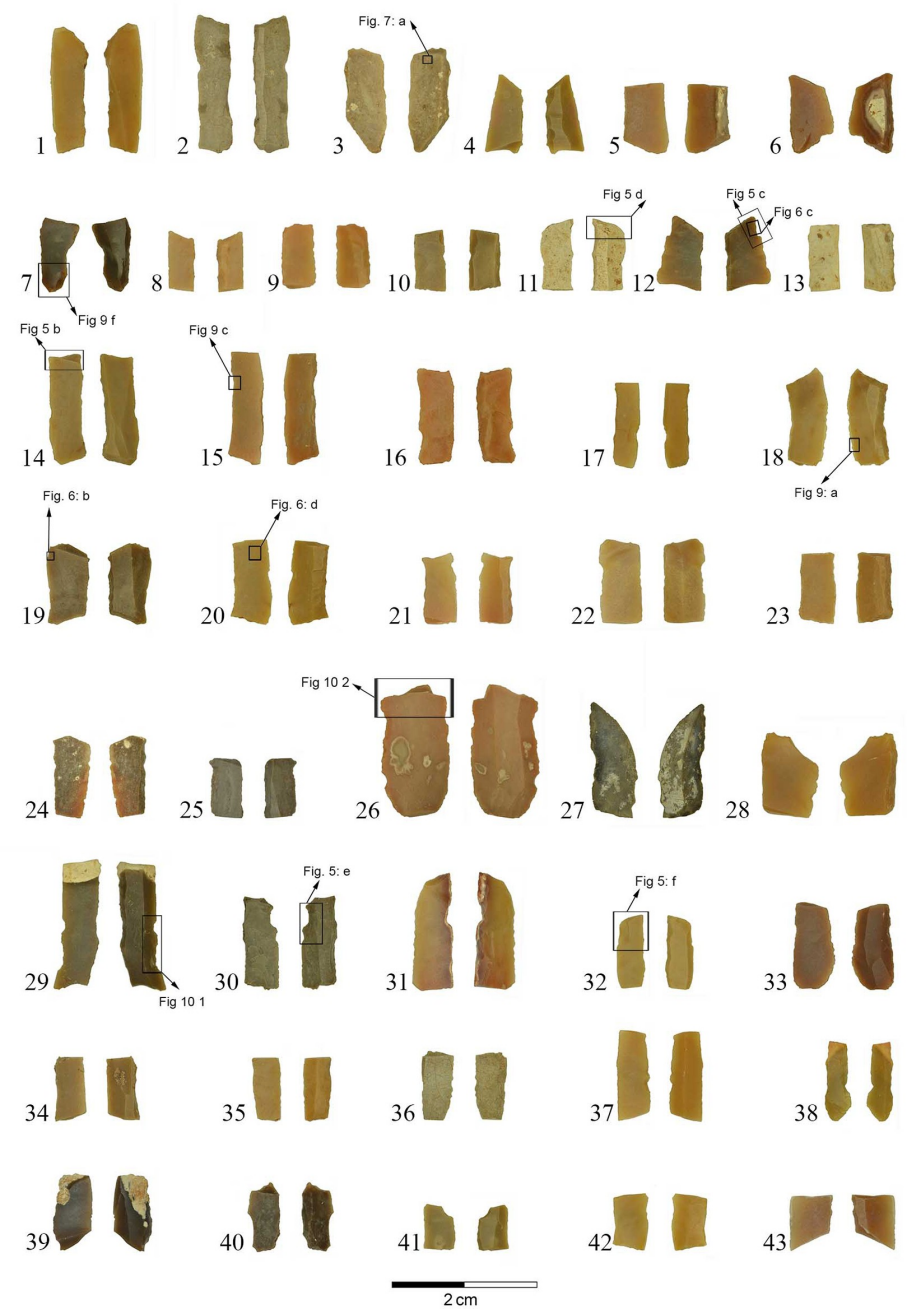
Microliths from Locus 5. (1–24) Geometric microliths. (25–43) Non-geometric microliths. Locations of detailed photos and micrographs are indicated, with references to Figs 6, 7, 9 and 10.

The majority of the tools (N = 119) found in Locus 5 are microliths (74.8%), similar to other samples at the site where they form ca. 80% of the tools (Table 3, Fig 2). The microliths were subjected to microscopic use-wear analysis.

**Table 3:** Breakdown of the tools from Locus 5.

Type	N	%
**Non-microlithic tools**		
Scrapers	1	0.8%
Burins	1	0.8%
Multiple tools	1	0.8%
Notches and denticulates	2	1.7%
Heavy duty tools	2	1.7%
Retouched flakes	12	10.1%
Retouched blades	9	7.6%
Varia	2	1.7%
**Sub-total**	**30**	**25.2%**
**Microliths**		
**Non-geometric microliths**		
Alternately retouched bladelets	4	3.4%
Pointed backed bladelets	1	0.8%
Obliquely truncated bladelets	1	0.8%
Retouched/backed bladelet fragments	32	26.9%
**Sub-total**	**38**	**31.9%**
**Geometric microliths**		
Straight truncated and backed	1	0.8%
Rectangles	2	1.7%
Trapeze/rectangles	1	0.8%
Trapezes	4	3.4%
Asymmetrical trapeze A	1	0.8%
Straight truncated and backed fragments	18	15.1%
Obliquely truncated and backed fragments	24	20.2%
**Sub-total**	**51**	**42.9%**
**Total microliths**	**89**	**74.8%**
**Total assemblage**	**119**	**100.0%**

Based on the type lists commonly used for the typology of microliths in the Levant [7,9].

In general, the microliths were mostly produced on narrow and thin bladelets averaging 5.4 mm in maximum width (s.d. 1.6). A straight profile and parallel lateral edges characterize the microliths, while curved and twisted blanks are rare (4.5%). Geometric microliths (42.9%, N = 51; Fig 2 1–24) are more common than the non-geometric types (31.9%, N = 38; Fig 2 25–43). A wide variety of retouch types was used for shaping the microliths, including abrupt, semi-abrupt, fine, inverse, alternate, mixed and bipolar; however, backing and truncating by abrupt retouch are the most common (77.5%, N = 69). These characteristics are common for other assemblages of microliths at the site.

### 2.2 Use-wear analysis and experimentation

In order to characterize the versatile use of microliths, we employed the analytical framework of use-wear analysis integrated with experiments using microliths. The analysis of microliths (N = 82, available at the time of research) from Locus 5 incorporated low and high-power observations. For the low-power analysis we used two stereomicroscopes (Nikon SMZ 745T and Zeiss Discovery V8, at magnifications of up to 80x (to document the morphometrics of edge removals and abrasions. Then, the high-power analysis was applied to observe micro-polishes and striations using a metallurgical microscope (Leica DM 1750M at magnifications 100-500x).

The first procedure of the analysis initiated in distinguishing the state of preservation by looking for traces resulting from post-depositional surface modification (PDSM) [51,52] including patination (mainly color alteration and glossy surface) and breakage. These traces are distinguished from use-wear mainly because they appear randomly or exhibit no clear patterns (such as directionality, morphology or polish texture) that may be the result of use. Then, traces associated with use were recorded in a contextual order on drawings; for the backed microliths, the studied pieces were positioned with the dorsal face up and the backed edge to the left. Sections with use-wear were defined as the potentially used area (PUA) and the location of the PUA was described accordingly. Finally, traces were photographed using the microscopes' cameras and in some cases we used the Z-stacking technique with Helicon Focus© software to obtain full-depth photos.

#### 2.2.1 Reconstructing projectiles through impact fractures

**Fig 3:**
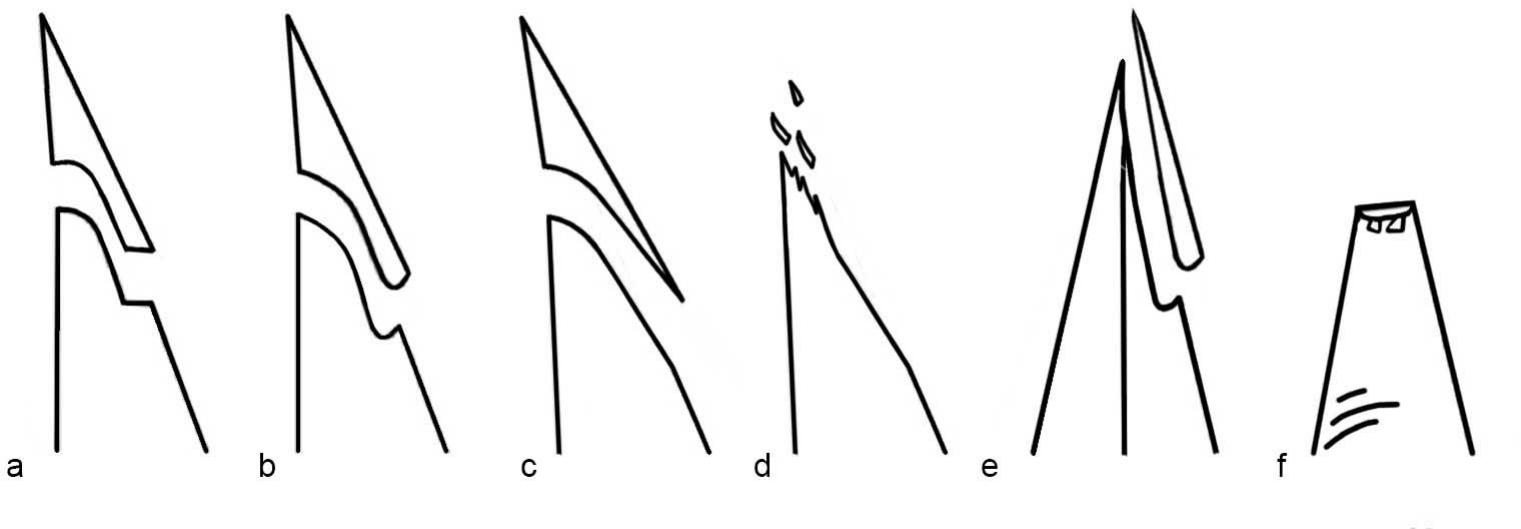
Schematic representation of DIF types considered in the current study. Side view showing the profile of (a) Step terminating bending fracture. (b) Hinge terminating bending fracture. (c) Feather terminating bending fracture. (d) Crushed fracture. (e) Dorsal view showing profile of burination on a sharp edge. (f) Ventral view showing two spin-off secondary cones initiating on a bending fracture.

In this study we relied on published analyses and experiments, demonstrating that Diagnostic Impact Fractures (DIFs) on microliths provide reliable identifications of projectiles [18,21,22,53–57]. Nonetheless, we used this information with caution, while considering the questions raised regarding the interpretation of DIFs, suggesting that their formation may be the result of various mechanisms and that they may differ in characteristics (shape, location on the blanks and directionality) in relation to the morphometrics of the studied blanks [51,58–61]. We therefore relied solely on studies of microliths and not of larger and less delicate types of tools. We are also aware that a single fracture on a microlith is not always enough for reconstructing projectiles [58,62], and in this analysis we applied the broad approach considering DIFs as well as linear features and hafting traces. A search for residue was also conducted throughout the analysis however with no positive results. Yet considering that the features other than DIFs were rare in the assemblage, we nonetheless considered DIFs as a critical indication for projectile use, as the study of microliths’ fractures is the main source of information. To confront this difficulty, and based on studies defining the types of fractures [22,53,54,63–68], the DIFs were strictly defined and we address four types of DIFs, especially relevant to the study of the ND microliths, all of which were documented in the low-power stage:

1. Step, feather or hinge-terminating bending fractures: the fracture initiates in a smooth surface which lacks a negative of a bulb and terminates in an abrupt angle (step) or smooth, moderate slope (feather), or an abrupt, curved edge angle (hinge). These fractures are usually created on the ventral face, but occasionally may appear transversal at the distal edge and rarely on the dorsal face (Fig 3A–3C).
2. Crushed fracture: a fracture composed of multiple, overlapping small fractures. This type may develop on both faces (Fig 3D).
3. Burination: a longitudinal burin-like fracture, usually propagating along the lateral edge (Fig 3E).
4. Spin-off fracture: a secondary cone fracture originating on the surface of a bending fracture and may appear more frequently on the ventral face than on the dorsal face (Fig 3F).

In addition, following previous micro-wear studies using the high-power technique [18,22,62,64], the microscopic linear impact traces, termed as MLIT by Moss [69], which are streaks of polish and striations that run along the direction of impact, directly associated with the DIFs, were also documented. Traces assumed to be the result of hafting were also recorded, including edge removals and characteristic polishes, however these were found in very low frequencies (five tools only, see below).

#### 2.2.2 Reconstructing the non-projectile functional aspect of microliths

The use-wear study was based on the protocol of use-wear analysis, looking first at the traces on the macro-scale and then at the micro-scale. We used a list of attributes for characterizing the wear features [70,71] and compared them to the reference collection of experimental tools.

The first stage included the study of the characteristics of edge removals, which on the macro-scale provide information regarding the hardness of the worked material, but also regarding the kinetics of the action and motion [72,73]. For edge removals, special attention was given to the scars' initiation (cone or bending), termination (for example, feather, step, hinge or snap) and distribution patterns (for example, close and run together, overlapping) [74,75]. Indications of motion and action, specifically relevant to the finds in the analysis of the ND microliths, are detailed in Table 4.

**Table 4:** General characteristics of the use-wear used to determine the method by which tools were used–the motion and then the action, relevant to the current study.

Motion	Transversal			Longitudinal
Action	Scraping	Shaving	Filleting	Sawing—cutting
Position of the tool in relation to axis of motion	Perpendicular		Perpendicular-oblique	Parallel
Angle of tool against the worked material	>45º	≤45º	≤45º	*ca*. 90º
**Macro-wear distinctions**				
Orientation of edge removals in relation to the edge of the tool	Perpendicular			Oblique
Location of edge removals	Opposite to main face in contact			Alternating (both faces) or on one face
**Micro-wear distinctions**				
Orientation of polishes and striations in relation to the edge of the tool	Perpendicular			Parallel
Location of polishes and striations	Main face in contact			Both faces

On the micro-scale, traces may provide additional information about the activity and specifics of the worked materials [76]. We used a series of attributes employed in use-wear analysis protocols [70], addressing texture, reflectivity, distribution pattern and topography for describing polishes and features associated with it (such as rounding of surface, striations, pitting). For the final functional reconstruction we primarily rely on cases where a distinct correlation between the macro and micro-wear was found. In cases where one of these aspects was missing (only macro-scale edge removals with no micro-scale polish was found), the interpretation was restricted according to the available information, to the hardness of the worked material (hard or soft, usually inferred through the size and shape of the edge removals), or to the motion by which the tool was used (cutting, scraping or shaving, also inferred through the shape, distribution and directionality of the edge removals).

#### 2.2.3 Reconstructing the aspect of grip

Another important component considered in the analysis procedure for the reconstruction of the mode by which tools were used is the issue of grip method. Specific type of wear is produced by the contact of the insert with a haft, including the adhesive material or binding material, or by the contact with a bear hand or a wrapping. These traces form in various conditions, depending on the method by which the tool was used, and the most indicative aspect of these traces is their location in relation to the use-wear. A separate list of attributes was used for defining wear produced through grip arrangements [71]. Although grip-related traces include several types (polish, rounding, smoothing, edge removals and linear features), in the current study wear associated with hafting was detected for only seven tools, and the most indicative type of wear is defined as ‘bright spots’ which is a localized patch of polish resulting usually from the contact with a haft or a haft with adhesive material. Only one tool showed a combination of bright spot and edge removals, which will be discussed in detail below.

#### 2.2.4. The reference collection of experimental tools

**Fig 4:**
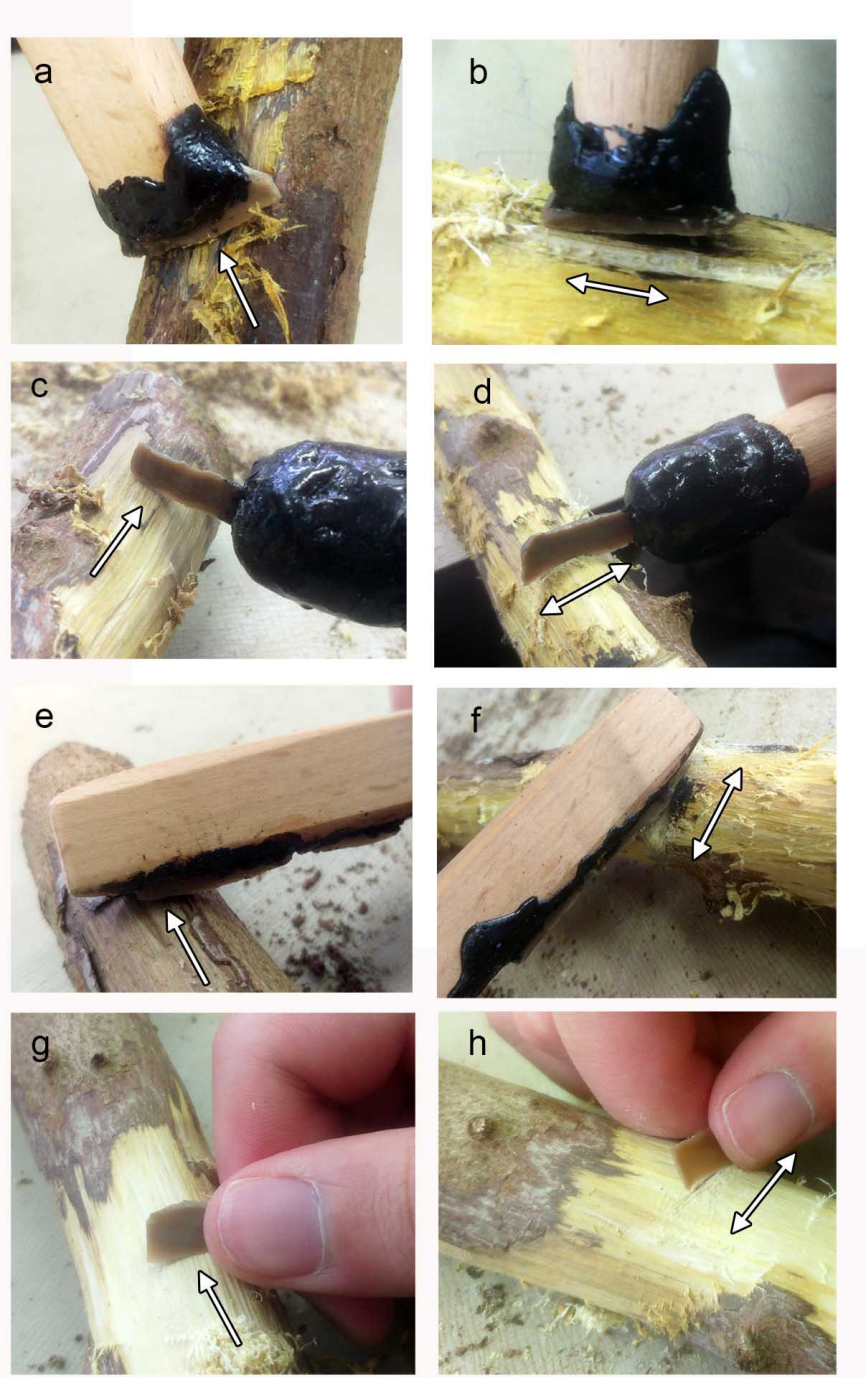
Experiments using various hafting arrangements and hand-held microliths for wood working. (a) Terminal-transversal hafting used for shaving bark and wood along the fibers. (b) Terminal-transversal hafting used for cutting wood along the fibers. (c) Terminal-longitudinal hafting used for shaving bark and wood along the fibers. (d) Terminal-longitudinal hafting used for cutting bark and wood perpendicular to the fibers. (e) Lateral-longitudinal hafting used for shaving bark and wood along the fibers. (f) Lateral-longitudinal hafting used for cutting bark and wood perpendicular to the fibers. (g) Hand-held microlith used for shaving bark and wood along the fibers. (h) Hand-held microlith used for sawing bark and wood perpendicular to the fibers. All the tools were used for 1000 strokes. Arrows indicate the direction of motion.

The functional reconstruction for tools that were defined as non-projectiles was based on a reference collection of experimental microliths bearing diagnostic traces produced through various tasks. A group of 23 bladelets were used for cutting cereals (N = 4, semi-dry wild barley), perforating shell (N = 2, *glycymeris*), bone (N = 3), wood (N = 3) and stone (limestone: N = 1, beach rock: N = 1), defleshing meat off bone (N = 3), meat filleting (N = 2) and cutting (N = 2) and shaving bone (N = 2). Additional 11 tools were used for working wood as this was one of the major functions identified for the microliths at ND (see below). In this set, the purpose was not only to replicate wood working traces, but also to examine specific aspects associated with the use of microliths, specifically the use of hafted compared to hand-held microliths. The goal was also to characterize the efficiency of the tools in working a relatively resistant hard material, assuming the experiment would shed further light on various aspects of the working process and possible need for adjustments and re-tooling. Although there are multiple possibilities for assembling composite microlith-equipped tools, we selected three basic hafting modes as well as three hand-holding positions of the microliths, and tested them on hard wood. The worked wood branches are of *Laurus nobilis* cut by the Israel National Parks Authority during trail clearance activity in the Mount Carmel forest near the University of Haifa. The wood was relatively fresh, with the bark, collected a few days after cutting; the microliths were produced from a local flint outcrop of Nahal Galim in Mount Carmel (Shamir Formation), with dimensions similar to those found in Locus 5 (Fig 4).

We examined two tasks (Fig 4), shaving the wood and sawing it, and each tool was used to a total of 1000 strokes. Hafted microliths were used in three different hafting arrangements:

1. Terminal-transversal hafting position where the microlith is placed at the end of the haft with its long axis perpendicular to the long axis of the haft (Fig 4A and 4B);
2. Terminal-longitudinal hafting position where the microlith is placed at the end of the haft with its long axis parallel to the long axis of the haft (Fig 4C and 4D);
3. Lateral-longitudinal hafting position where the microlith is placed along the side of the haft with its long axis parallel to the long axis of the haft (Fig 4E and 4F),

The hafts were made of hard beech (*Fagus*) wood, 30 cm in length and 2 cm in diameter except for the terminal-longitudinal mode which was narrower at the tip (1.5 cm) to allow the grip of the narrow longitudinally inserted microlith. Grooves were sawn at the distal end for two of the hafts (terminal-transversal and terminal-longitudinal arrangements) and one on the side (lateral longitudinal arrangement). The microliths were glued using bitumen and in the case of the terminal-longitudinal arrangement used for sawing we added a string to fasten the bladelet more firmly because the bladelet kept moving out of the bitumen during the work. Microliths held by hand were also used for 1000 strokes against the wood, for cutting and shaving (Fig 4G and 4H).

## 3. Results

The use-wear analysis of microliths (N = 82, Table 5) resulted in a high frequency of items with diagnostic traces (N = 52, 63%) indicating the high use-rate of microliths. Twenty-two items (27%), among them two complete geometric types, exhibit some form of damage which could not be associated with utilization and therefore defined as not diagnostic. Three showed no traces and five exhibited a high rate of post-deposition surface modification (PDSM) that prevented clear view of the flint surface and therefore were not further analyzed. Detailed information about the analyzed specimens and results appear in supporting information.

**Table 5:** The results of the use-wear analysis for the microliths from Locus 5.

Type		N	%
**Indicative use-wear**	Projectile	34	65%
	None-projectile	18	35%
**Sub-total**		**52**	**63%**
	None-diagnostic	22	27%
	No traces	3	4%
	PDSM	5	6%
**Sub-total**		**30**	**37%**
**Total**		**82**	**100%**

### 3.1 Projectile microliths

**Fig 5:**
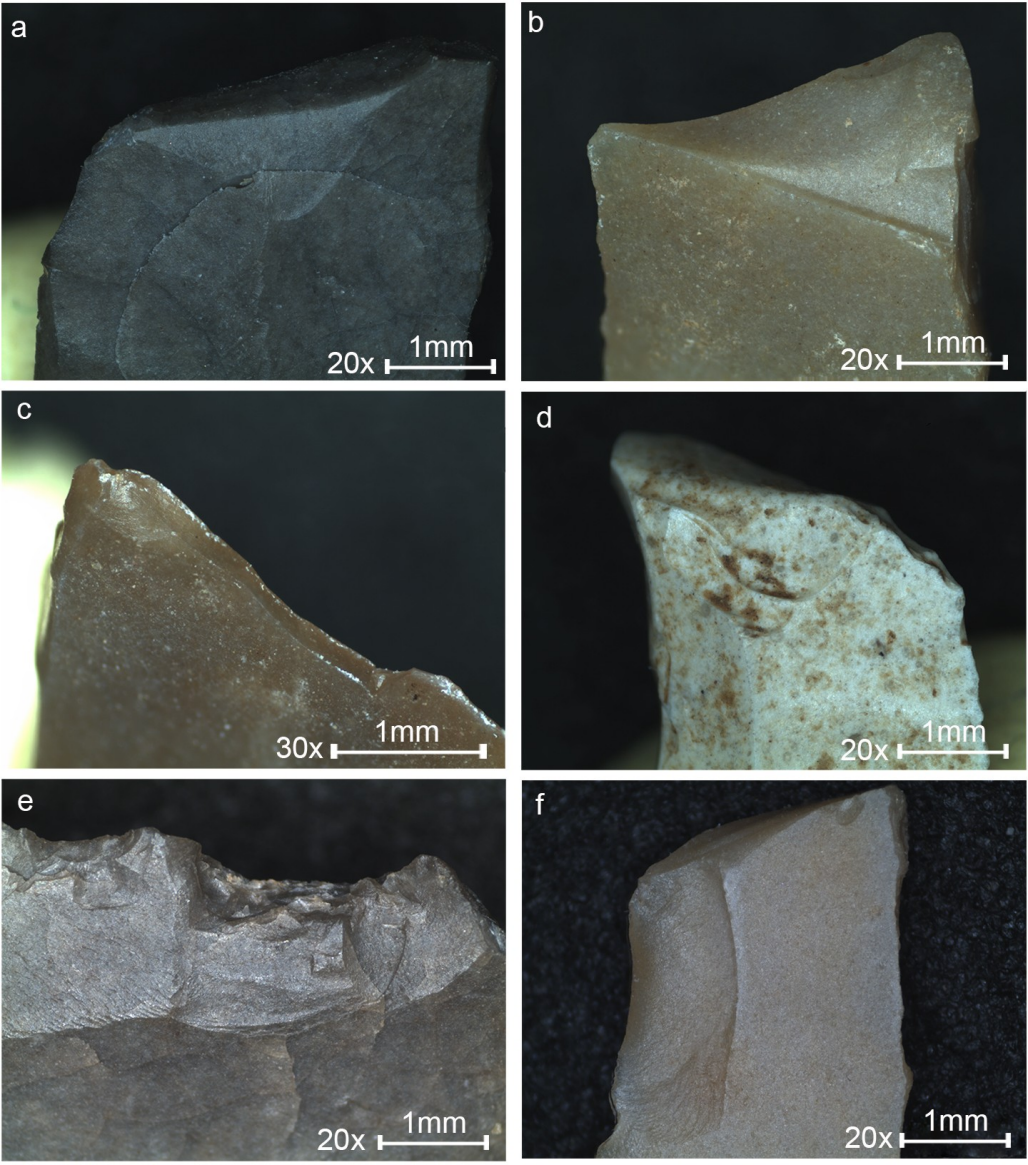
DIFs observed on the ND microliths. (a) Feather terminating bending fracture. (b) Step terminating bending fracture, see also Fig 2 14. (c) Burination, see also Fig 2 12. (d) Hinge terminating bending fracture, see also Fig 2 11. (e) Crushing, see also Fig 2 30. (f) Spin-off fracture at distal and step terminating bending fracture on left lateral, see also Fig 2 32. Original magnification is indicated near the scale bar.

Many microliths (n = 34, 65% of the microliths with diagnostic use-wear) were found to bear DIFs and accordingly defined as projectile inserts (Table 6, Fig 5).

**Table 6:** Frequency of microliths with DIFs.

Use-wear	Geometric	Non-geometric	N	%
Bending fractures	12	8	20	58
Crushing	2	2	4	12
Burination	2	2	4	12
Spin-off	2		2	6
Bending fracture and burination		1	1	3
Bending fracture and spin-off	1	2	3	9
**Total**	**19**	**15**	**34**	**100**
**%**	**55**	**45**	**100**	

The various types of DIFs were observed for both geometric (N = 19) and non-geometric (N = 15) microliths. Most microliths were interpreted as projectile tips (N = 30), exhibiting the DIFs at the proximal (N = 8) but more frequently at the distal end (N = 15). Several exhibit a DIF on both ends (N = 4), while others (N = 7) have DIFs on the sharp lateral extending perpendicular or obliquely to the long axis of the insert. The latter may be interpreted as transversal points or as barbs.

**Fig 6:**
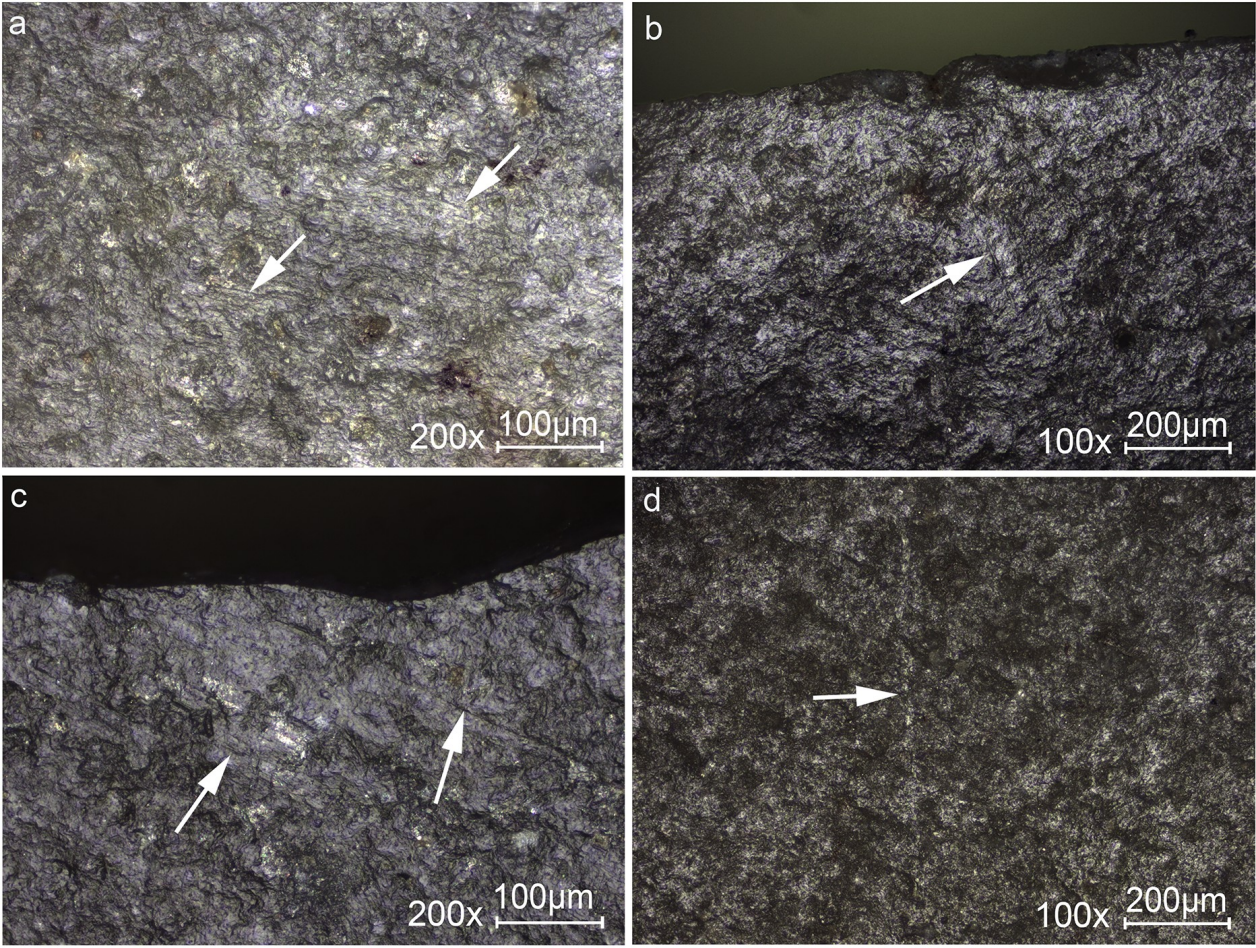
Micrographs showing linear traces associated with DIFs. (a) A cluster of fine striations, see also Fig 2 3 for the location of the traces on the tool. (b) Isolated sterea, see also Fig 2 19 for the location of the traces on the tool. (c) Striations extending oblique to a burination DIF, see also Fig 2 12 for the location of the traces on the tool. (d) Isolated streak of polish extending away from the impact fracture, see also Fig 2 20 for the location of the traces on the tool. Original magnification is indicated near the scale bar.

Micro-wear associated with the DIFs observed through high-power analysis was detected only on four of the microliths. These include striations (Fig 6A–6C) or a streak of polish (Fig 6D) extending away from the DIF visible on the ventral face. Two of these are transversal tips.

### 3.2 Non-projectile microliths

#### 3.2.1. The results of the experiments

**Fig 7:**
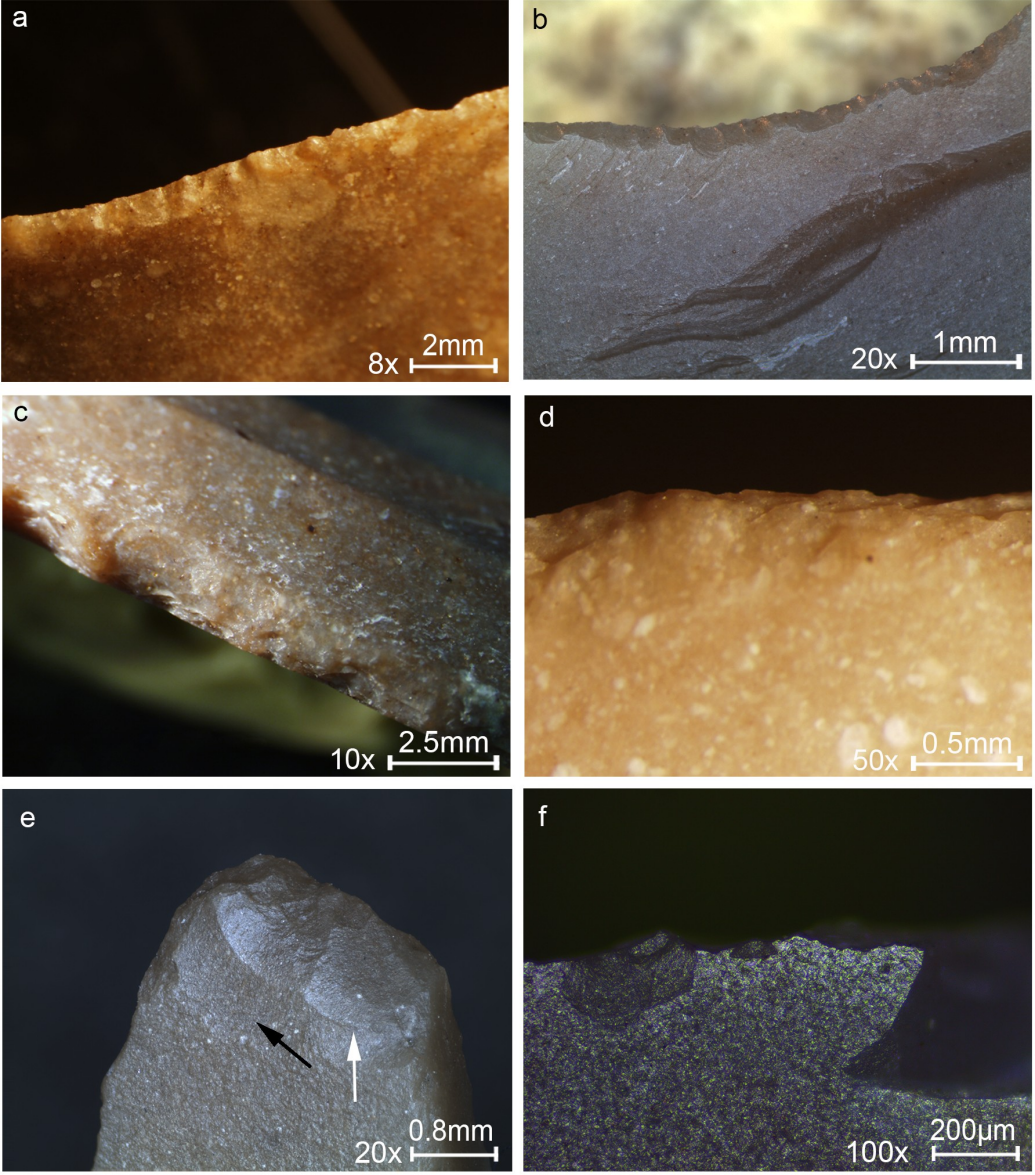
Key examples of non-projectile use-wear observed on experimental tools. (a) Sawing wood for 60 minutes showing close and overlapping edge removals along the edge, usually with cone initiation and step termination, extending in two directions oblique or perpendicular to the working edge indicating the motion of the tool. (b) Scraping wood for 45 minutes showing a convex edge resulting from prolonged contact with the wood at the same spot, with edge removals close and run together, with cone initiation and feather termination, an axis perpendicular to the edge and creating an almost abrupt edge. (c) Sawing bone for 10 minutes showing an irregular shape as a result of the massive damage, edge removals are close, vary in size with a crushed initiation and feather termination. (d) Scraping bone for 38 minutes showing a section of edge removals, overlapping, stepped, with step termination. (e) Drilling dry bone for 18 minutes showing fractures with an oblique axis that indicates the rotational motion with slight crushing at the tip resulting from the downwards pressure. (f) Defleshing for 2 hours showing tiny edge removals associated with a weak polish produced by the contact with flesh and periosteum spreading in a scintillation pattern along the edge. Original magnification is indicated near the scale bar.

**Fig 8:**
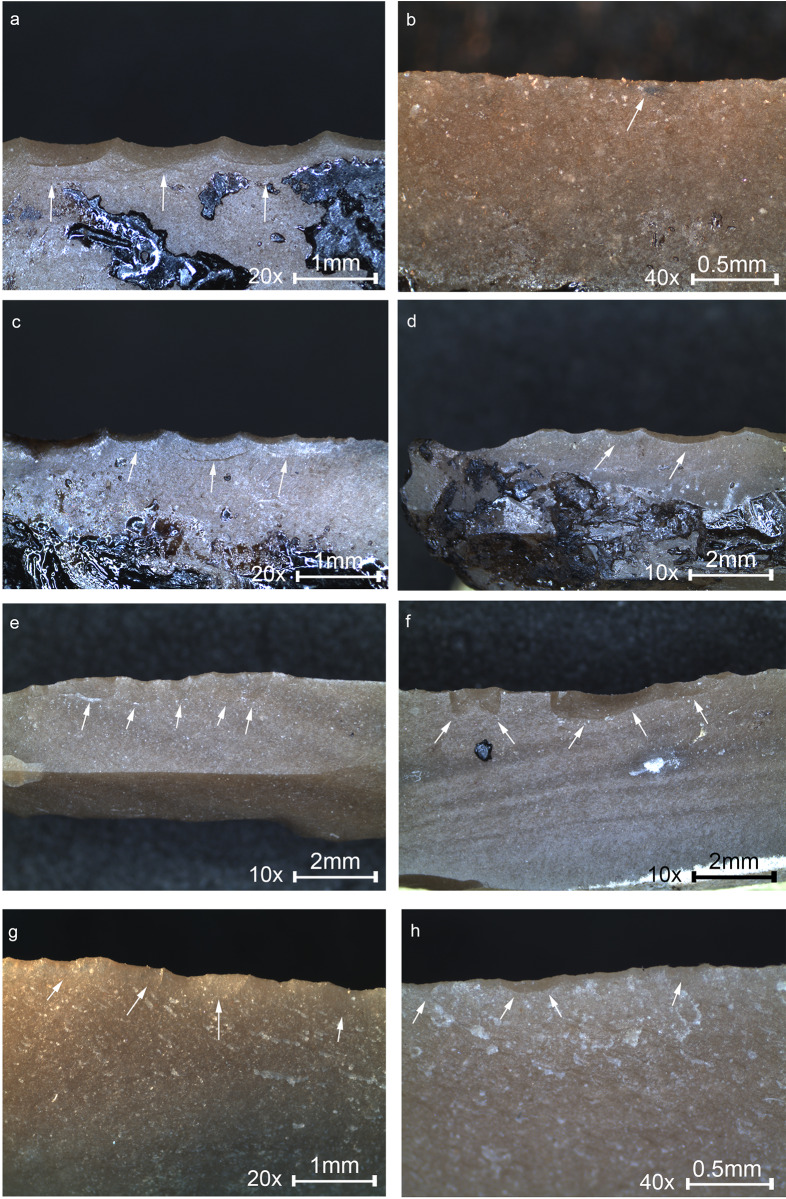
Macrographs showing macro-wear on experimental tools from the experiments in this study. (a) Terminal-transversal hafting, shaving wood: edge removals close and run together, with bending initiation and step termination (marked by arrows), located at the middle part of the tool. (b) Terminal-transversal hafting, sawing wood: tiny edge removals with cone initiation and step termination in a cluster, overlapping and with an axis oblique to the working edge (an example marked by an arrow). (c) Lateral-longitudinal hafting, shaving wood: edge removals of the same type as in macrograph a, but smaller in size. (d) Lateral-longitudinal hafting, sawing wood: heavy damage of edge removals of different size and shape. (e) Terminal-longitudinal hafting, shaving wood: edge removals separated and run together along the working edge, invasive, with bending initiation and step termination, extending in an axis perpendicular to the edge, located at the un-hafted upper part of the tool. (f) Terminal-longitudinal hafting, sawing wood: edge removals of different size and shape, extending in two directions in an axis oblique to the edge. (g) Hand-held, shaving wood: tiny edge removals, some with bending initiation and with a feather termination extending in a more or less perpendicular axis to the edge. (h) Hand-held, sawing wood: tiny edge removals of different size and shape extending in two directions in an axis oblique to the edge. Original magnification is indicated near the scale bar.

The experiments using the hafted and hand-held microliths provided unique insights for understanding the high performance and limitations in using microlithic tools. Fig 7 shows key examples of use-wear with distinct traces relevant to understanding the functioning of microlith and the formation of the traces. Fig 8 shows the results of the set of experiments in wood-working, specifically relevant to one of the major tasks identified at ND.

In general, all the tools were highly effective and some of them were still totally functional at the end of the experiments (after 1000 stroked in the wood working experiments). It was obvious that the hafting enhances the efficiency of the tool, which can be used more conveniently and effectively compared to the hand-held tools. Hafting also provided better control on the motions and force application. For the hafted tools, two specific limitations were observed, both of which are the result of the narrow working edge protruding from the haft. The first concerns the angle of work, which is the most outstanding limitation, associated with the thickness of the haft at the joint, where the insert is glued. If the haft is too thick at this point the tool cannot work in a low angle because the protruding edge of the insert is very small (a few mm). Therefore, the haft should be held in a relatively upright angle to prevent the contact of the mastic and haft with the worked material (Fig 4A). The second limitation concerns the ability to penetrate into the worked material in the case of sawing (Fig 4B). The depth of the cut is limited to the width of the microlith, therefore it is possible to cut only few millimeters into the worked material.

Another important aspect identified during the experiments concerns the hafting mode. The terminal-longitudinal hafting required an additional adjustment to fix more firmly the long protruding bladelet (Fig 4C and 4D). The bitumen broke during the work and the microlith fell out of the haft. This happened again even after adding string binding. Therefore, it is evident that longitudinal hafting requires a different, more reliable fixation (perhaps a different formula for glue or binding). Given these results, this hafting mode is more effective for sawing more deeply into the worked material, as opposed to the terminal-transversal and lateral-longitudinal modes.

The use-wear observed on the experimental microliths provides valuable information which may be used for reconstructing aspects associated with motion, action and grip:

1. In general, orientation and location of edge removals are distinctively diagnostic to the motion and action (Table 4); the transversal scraping and shaving produced scars in an orientation perpendicular to the edge located opposite to the surface in contact (Fig 7B, 7D, Fig 8A, 8C and 8E) and the longitudinal sawing produced edge removals in varying orientations, indicating the bidirectional motion of the tool, located on both faces of the tool (Fig 7A and 7C, Fig 8D, 8F and 8H). The wood and bone sawing in an angle of 90º produced scars with cone initiation and feather termination (Fig 7A and 7C, Fig 8B, 8D, 8F and 8H), less distinct in shape and size. This pattern is clearly distinguished from wear pattern produced by the wood shaving in an angle of <45º, which is consistent in our experiments, exhibiting the invasive bending initiation and step termination scars close and run together (Fig 8A, 8C, 8E and 8G). Scraping in an angle >45º produced scars positioned in a more abrupt angle (Fig 7B and 7D).
2. The distribution pattern of the edge removals is more distinct for tools used in a transversal mode. Wood scraping produced scars close and run together (Fig 7B), similar to the wood shaving (Fig 8A, 8C, 8E and 8G) and bone scraping produced overlapping stepped scars (Fig 7D), a more massive damage reflecting the hardness of the bone.
3. The degree of damage indicates the hardness of the worked material, with some exceptions, as worked materials may be treated in different ways. For example, in our experiments one of the sawing sessions was done parallel to the wood fibers (Fig 4B) causing a mild alteration (Fig 8B), but sawing perpendicularly to the fibers formed a more massive damage, altering the shape of the edge more massively (Fig 7A). Bone produced more massive damage as it is harder than wood (Fig 7C and 7D), distinctively different from wood working, both by cutting and scraping (Fig 7C and 7D and Fig 8). A distinct change in the shape of the edge was created (a convex edge) by scraping in an angle ≥45º (Fig 7B), a feature not observed by shaving (yet, it is possible that a longer use of the tool may create a more massive change in the shape of the edge). Scraping may therefore also result in frequent re-tooling compared to shaving.
4. Hafted tools result in a more massive damage because a haft allows the application of greater force and the damage is accordingly more massive (Fig 8A–8F). Hand held tools exhibit faint signs of use (Fig 8G and 8H), and may therefore be harder to identify in an archaeological assemblage.
5. A haft also allows a more accurate work therefore creating a more organized and distinct wear pattern. The distribution of the wear however, may be used to distinguish the method of grip as use-wear is produced only in the contact area. For example, wear was produced at the upper part of the tool, at the un-hafted part in the terminal-longitudinal hafting arrangement (Fig 8E). The same location may be expected for a tool held by hand at one side, however for hand-held tools the small size of scars and low intensity of damage is more distinct. In comparison, wear was produced at the middle part of the tool for the transversal task using the terminal-transversal hafting arrangement (Fig 8A and 8B).
6. The drilling experiments produced clear damage reflecting the vertical penetration combined with the rotational motion of the dill point. The rotation produced scars oriented perpendicular or oblique to the axis of the tool (Fig 7E, black arrow) and the vertical force produced scars oriented parallel to the axis of the force (Fig 7E, white arrow).

#### 3.2.2. The results of use-wear analysis of the archaeological microliths

**Fig 9:**
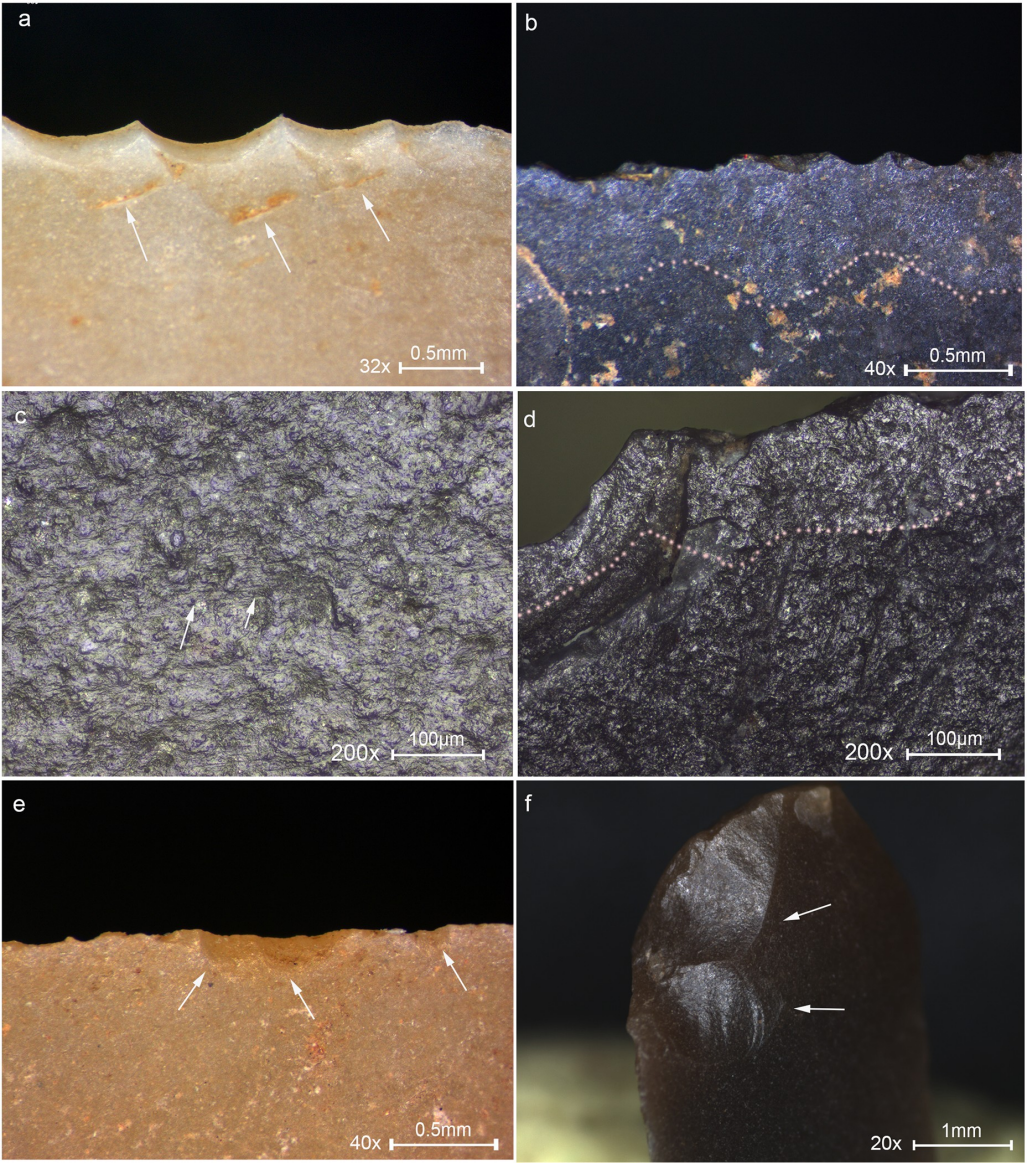
Macro and micrographs of non-projectile use-wear observed on the Locus 5 microliths. (a) Edge removals close and run together, with bending initiation and step termination with an axis oblique to the edge indicating the oblique position of the haft, restricted to the upper part of the tool, interpreted as wood shaving tool used hafted in a terminal-longitudinal arrangement, see Fig 2 18 for the location of the traces on the tool. (b) Invasive band of faint polish, marked by the dotted line, interpreted as transversal filleting of meat. (c) Rough, dull polish with multiple longitudinal fine striations shown by the arrows, interpreted as cutting herbaceous plants with the intervention of dust particles, see Fig 2 15 for the location of the traces on the tool. (d) Band of faint polish marked by the dotted line developed to a low degree along the edge, interpreted as meat cutting, see Fig 2 29 for the location of the traces. (e) Tiny edge removals extending in two directions associated with meat cutting polish indicating bidirectional cutting. (f) Edge removals with cone initiation and feather termination extending obliquely-perpendicular to the axis of the tool, observed on the pointy edge of a microliths, indicating the rotational motion of the tool, interpreted as a drilling tool, see Fig 2 7 for the location of the traces on the tool. Original magnification is indicated near the scale bar.

The analysis detected a variety of functions other than projectiles (N = 18, 35% of the microliths with use-wear; Table 7), although to a lower degree, and of note is that most of them are on the geometric types (N = 13, 77%). Traces were observed on the macro-scale and on the micro-scale and indicate that tools were used in a variety of motions and actions, by exploiting mostly the long sharp lateral edge (Fig 9).

**Table 7:** Cross-tabulation of working motion and contact materials interpreted for non-projectile microliths.

	Meat	Herbaceous plant	Wood	Soft material	Material not diagnostic	Total	%
Sawing/cutting	3	1		1		**5**	**27**
Sawing and scraping					1	**1**	**6**
Shaving			8			**8**	**43**
Filleting	1					**1**	**6**
Drilling					1	**1**	**6**
Hafting only					2	**2**	**12**
**Total**	**4**	**1**	**8**	**1**	**4**	**18**	**100**
**%**	**22**	**6**	**44**	**6**	**22**	**100**	

Tools were used more frequently for transversal actions (scraping, shaving, filleting, 55%) in which the motion of the tool is perpendicular to the long sharp edge. This includes microliths used for shaving wood (Fig 9A), where the use-wear was also found restricted to the upper or lower part of the tool, similar to tools used in the experiments with a terminal-longitudinal hafting arrangement (Fig 8E). In our experiments (Fig 4C and 4D) this proved to be highly effective, but adequate hafting is crucial in this case as the high pressure applied in this position can easily move the insert out of the haft. This method overcomes the problem of using a thick haft, enabling the use a microlith for a variety of actions, both longitudinal and transversal, and application of deep cuts. Meat filleting (Fig 9B) is another function where tools were used transversally, identified by the distinctive polish in our experiment (Fig 7F).

Tools were also used in longitudinal actions (27%) in which the motion of the tool is parallel to the long sharp edge. These tools were used particularly against soft materials, including cutting herbaceous plants (Fig 9C) and meat (Fig 9D–9F), and one tool exhibiting minor damage of tiny scars in oblique orientation, is interpreted to have been used for cutting a soft material (Fig 9E). The functional reconstruction for this tool is therefore given with low certainty. Only one tool was used by the pointy distal end for drilling, exhibiting edge removals on the pointed edge, in orientations that indicate the rotational motion of the tool (Fig 9F), similar to the microliths used for drilling in the experiment (Fig 7E), yet it is impossible to determine the worked material for the ND tool as no polish was developed.

Microliths were used for working hard materials such as wood (N = 8, 44%) and for soft materials such as flesh The contact with a relatively hard substance such as wood produced quite heavy damage along the sharp lateral edge compared to the soft materials. The experiment in wood working showed that albeit this damage, the tools were still functional, however at some point a replacement of the insert was probably required. In comparison, microliths that were used for working with soft materials (N = 5), as in the case of meat and herbaceous plants, bear microscopic damage, with slight or no damage on the macro-scale. For these tools micro wear (polish and striations) was also observed. The use of these tools seems to have been relatively short as the cutting edge is intact and may still be used.

Worth mentioning is the microlith used for cutting herbaceous plants (Fig 9C). The wear observed is faint, including dull polish with multiple thin, shallow and long striations that run parallel to the working edge. This was not replicated in our experiments in this research, therefore the interpretation is given with low certainty. However, based on past experiments in cereal harvesting, these striations indicate the intervention of tiny particles, perhaps dust, that may indicate the use for cutting grasses. In this case, as for the other tools used for working with a soft material, the task was short and it seems that the tool was discarded although it was still useful.

### 3.3 Hafting

**Fig 10:**
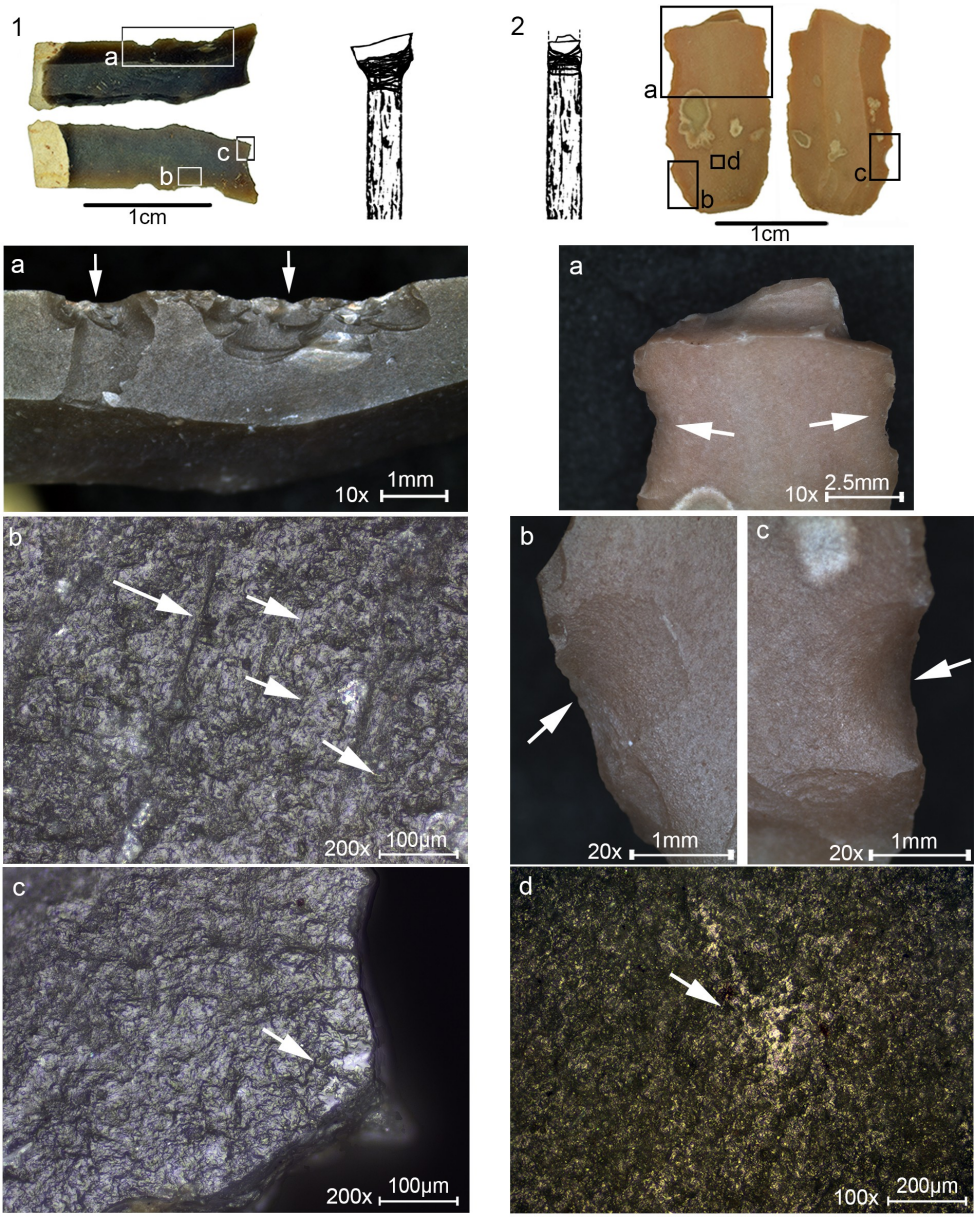
Microliths with traces associated with hafting and a reconstruction of the hafting arrangement. (1) Microlith showing multiple DIFs on the sharp lateral interpreted as a transversal point hafted in a terminal-transversal hating arrangement depicted in the drawing. (1a) Close view of the DIFs with arrows showing the direction of impact. (1b) Striations MILT observed on the ventral face opposite the DIFs indicating the direction of impact. (1c) A bright spot shown by the arrow interpreted to be produced by the contact with the haft and perhaps adhesive material. (2) A microlith with DIF at distal end interpreted as the hafted part of a point due to the presence of hafting traces at the lower part of the tool opposite the break. (2a) Close-up view at the bending fracture and opposing lateral notch-like damage of overlapping edge removals assumed to be produced due to binding. (2b) An isolated fracture with bending initiation and step termination assumed to be produced by the contact with a haft and perhaps binding. (2c) Another isolated fracture, opposite the fracture in Fig 2B, termed by Rots [71] sliced into scalar bending scar, assumed to be the result of the contact with the haft and perhaps binding. (2d) A bright spot resulting from the contact with the haft and perhaps adhesive material observed on the bulbar area. Original magnification is indicated near the scale bar.

Seven microliths have wear traces associated with hafting; four are projectiles (Fig 10 1), two microliths are the hafted segments with the presumed working edge broken (Fig 10 2), and another one was defined as a wood shaving tool. In our experiment we did not observe hafting traces therefore our interpretation is based on evidence published by Rots [71], with the location of the traces along the item being another leading evidence for reconstructing the hafting arrangement. The hafting traces found on the ND microliths are located at the lower part of the tools, however, considering that hafting traces are extremely localized, especially the bright spots, the reconstruction of the hafting arrangement should be taken with caution.

For tools interpreted as projectiles, the direction of the DIFs may be an indication to the location of the haft on the opposite side. In the case of a microlith interpreted to be a transversal point (Fig 10 1) the direction of the DIFs (Fig 10 1A shown by the arrows) correspond to the direction of the linear features (MILD) (Fig 10 1B shown by the arrows) indicating the direction of impact. A bright spot was observed opposite the DIFs (Fig 10 1C), on the edge of the retouched back, indicating that this part was in the haft. Combined with the characteristics of the DIFs it is possible to reconstruct the terminal-transversal hafting arrangement. For the rest of the three microliths exhibiting bright spots at the lower part of the tool, opposite to the DIFs, a terminal-longitudinal hafting arrangement is reconstructed.

Where hafting traces appear on both laterals and on the bulbar surface, as in the case of the hafted piece in Fig 10 2, a terminal-longitudinal hafting arrangement is suggested. This tool in particular exhibits edge removals that may result from pressure against binding material at the upper part of the tool (Fig 10 2A) as well as at a lower part, showing opposing isolated scars, one which is especially typical, termed by Rots [71] sliced into scalar bending fracture (Fig 10 2C).

## 4. Discussion and conclusions

Microliths have been studied for more than a century and their adaptive advantages in foraging lifeways have been widely evaluated. In correlation to the course of the development of the archaeological discipline, most of the advantages from past studies are discussed from their morphological and technological aspects [67,77–80]. These studies established a firm base portraying the hunter-gatherer societies of the Late Pleistocene as capable of developing and exploiting technologies to increase their adaptation in a way much different from that of former societies. Although the versatile use of microliths has been advocated before, this aspect is still scarcely addressed in the literature [18].

Our goal was thus to examine whether former analyses results of the versatile use of microliths, especially those indicating their exploitation in a large range of tasks [26], can now be considered as an integral part of this technological organization. If so, we should redefine the complexity and advantages of microlith use. We focused on a case study from the Levantine Epipaleolithic, which represents one of the most familiar cases of enhanced microlithization.

Toward a better understanding of the versatile use of microliths the current study focused on the functional aspects of microliths from one context at the GK site of Neve David. Microliths are the most abundant tools, comprising the hallmark of the GK, and the results support several past studies that show their use as weaponry tools [18,20–22,53,81]. The typical DIFs are the most common damage observed on the microliths. The Neve David Locus 5 assemblage includes mainly points and the use of composite tools is clearly implied also by the evidence of hafting. Looking at the types of microliths, there is no distinct functional difference between geometrics and non-geometrics; both groups were used as tips in composite projectiles (Table 5), yet a tendency to use geometric types is observed for non-projectile tools.

The numerous microliths bearing DIFs could be associated with the rich faunal assemblage retrieved at Neve David. Hunted game include mountain gazelle (*Gazella gazella*) and Mesopotamian fallow deer (*Dama mesopotamica*) that comprise most of the faunal assemblage and bear ample evidence of butchery and consumption [31,39]. Direct faunal evidence for the use of microliths as hunting tools is still lacking at Neve David and for the GK in general, but two gazelle bones bearing projectile injuries from the Early Natufian of el-Wad Terrace [82] and a lunate fragment embedded in a Natufian human vertebra in Kebara Cave [38] attest to the use of microliths as projectiles.

While the use of microliths as inserts in composite projectiles is well known and established, our major contribution however was aimed towards examining the extent of their functional range of use beyond projectile technology. The multi-function use of microliths has rarely been discussed, although when the full procedure of use-wear analysis was applied, the variety of functions became apparent; such was the case for microliths from several GK sites in Jordan [18] and for several Natufian sites [28]. Our results indicate that the Neve David microliths have been used for a variety of functions, such as sawing, scraping and boring. Butchery and especially wood working were the common functions detected after provisioning for projectiles. While butchered animal bones are abundant in Locus 5 and in the camp in general, macroscopic wood and other plant remains are hardly preserved at the site, emphasizing the importance of our use-wear results to assess plant exploitation and use in the GK. The rare evidence for wood craft found at the early Epipaleolithic site of Ohalo II, where a variety of objects were found on brush hut floors, including "pencil-like" shaved twigs /small branches [83], sheds light on wood use in the late Pleistocene Levant. Likely, microliths were used as inserts in composite tools but our experiments show that it is possible to work hard materials with microliths held by hand. The cutting of herbaceous plants may tentatively suggest plant gathering during the Middle Epipaleolithic, and considering that the archaeobotanical remains are still under processing for Neve David, this is an important result indicating gathering of plants at the site. This activity was extensively identified at Ohalo II, through the botanical remains of small-grained grasses, cereals, and sickle blades [84–91].

The results of our research indicate a highly versatile adaptive strategy in the Epipaleolithic of the Levant, where the microliths were used as inserts within composite tools for a variety of functions. Furthermore, we can illustrate a case in which a set of microliths put in a "pocket" and transported by mobile hunter gatherers, as part of residential or logistical mobility patterns, could have enabled them to maintain as well as to create at their will, composite tools for a wide range of functions. In other words, it provided them with a flexible solution to a variety of needs they may encounter during their travel throughout the landscape with a reduced cost. We did not recognize reuse of the same piece for different functions. This may support the notion of high microlith availability in which inserts replacement is preferable than their rejuvenation.

Our results demonstrate yet again the complexity of microlith function in the Levantine Epipaleolithic, indicating that flexibility in the use of tools was an inherent aspect within these industries. Former studies emphasized two main advantages in addressing the technological organization of microlithic industries. The first focused on the advantages of production in terms of raw material economy and the transportability of the products over the landscape. The second, which corresponds with the first, focused on the haftability, enabling maintainable and reliable toolkits for foraging hunter gatherers. Our results confirm the clear presence of a third advantage, addressing the microliths' highly versatile use for a variety of functions. We argue that the combination of these three aspects sheds new light on the high degree of versatility of Levantine Late Pleistocene hunter gatherers, and presents more clearly the technological choice of microlithization. While the current results address the Levantine record, we expect this phenomenon to be much wider both in space and time, as the scarce published works regarding microlith function other than projectile technology already suggest.

## Supporting information

S1 Data (DOCX)
