# Supplementary material for: Versatile use of microliths as a technological advantage in the miniaturization of Late Pleistocene toolkits: The case study of Neve David, Israel
Source: PLoS One. 2020 Jun 3;15(6):e0233340. doi: 10.1371/journal.pone.0233340 (PMC7269238; doi:10.1371/journal.pone.0233340)
Supplement: S1 Data — (DOCX) [file pone.0233340.s001.docx]

|  | **NO.** | **TYPE** | **sub TYPE** | **PDSM rate** | **PUA** | **FUNCTIONAL RECONSTRUCTION** | **Traces type** | **Hafting traces** |
| --- | --- | --- | --- | --- | --- | --- | --- | --- |
| 1 | 1154-V21b-1 | geometric | Rectangle | low | not diagnostic | not diagnostic | not diagnostic |  |
| 2 | 1409-W21a-2 | geometric | Trapeze | low | not diagnostic | not diagnostic | not diagnostic |  |
| 3 | 1409-W21a-3 | geometric | Backed and straight fragment | high | not diagnostic | not diagnostic | not diagnostic |  |
| 4 | 1409-W21a-4 | geometric | trapeze | low | proximal end | projectile tip | DIF (spin-off) |  |
| 5 | 1409-W21a-5 | geometric | Backed and oblique fragment | low | proximal end | projectile tip | DIF (bending feather) |  |
| 6 | 1409-W21a-6 | non geometric | Alternately retouched bladelet | low | distal end | projectile tip | DIF (burination) |  |
| 7 | 1409-W21a-7 | non geometric | Retouched/backed fragment | low | sharp lateral | cutting meat | edge removals and polish |  |
| 8 | 1149-W21a-1 | non geometric | Alternately retouched bladelet | high | PDSM | PDSM | PDSM |  |
| 9 | 1149-W21a-2 | geometric | trapeze | low | not diagnostic | not diagnostic | not diagnostic | Edge removals and bright spot |
| 10 | 1149-W21a-3 | geometric | Backed and oblique fragment | low | right lateral | transversal projectile tip | DIF (crushing) and striations | bright spot |
| 11 | 1149-W21a-4 | geometric | Rectangle | low | sharp lateral | shaving wood | edge removals |  |
| 12 | 1149-W21a-5 | geometric | Backed and oblique fragment | low | sharp lateral | cutting herbaceous plants | edge removals, polish, striations |  |
| 13 | 1149-W21a-6 | geometric | Backed and oblique fragment | low | sharp lateral | shaving wood | edge removals |  |
| 14 | 1149-W21a-7 | geometric | Backed and oblique fragment | high | PDSM | PDSM | PDSM |  |
| 15 | 1149-W21a-8 | geometric | Backed and oblique fragment | low | distal end | projectile tip | DIF (bending feather ) and striations |  |
| 16 | 1149-W21a-9 | non geometric | Retouched/backed fragment | low | not diagnostic | not diagnostic | not diagnostic |  |
| 17 | 1149-W21a-10 | geometric | Backed and oblique fragment | low | sharp lateral | transversal projectile or barb | DIF (crushing ) and striations |  |
| 18 | 1149-W21a-11 | geometric | Backed and straight fragment | high | distal end | projectile tip | DIF (bending feather) |  |
| 19 | 1149-W21a-12 | geometric | Backed and straight fragment | low | sharp lateral | shaving wood | edge removals |  |
| 20 | 1149-W21a-13 | non geometric | Retouched/backed fragment | high | distal end | projectile tip | DIF (burination) |  |
| 21 | 1149-W21a-14 | geometric | Backed and oblique fragment |  | sharp lateral | shaving wood | edge removals |  |
| 22 | 1149-W21a-15 | non geometric | Retouched/backed fragment | low | distal end | projectile | DIF (bending step and spin-off) |  |
| 23 | 1149-W21a-16 | geometric | Backed and straight fragment | low | not diagnostic | not diagnostic | not diagnostic |  |
| 24 | 1149-W21a-17 | non geometric | Retouched/backed fragment | low | both ends | projectile | DIF (bending step and spin-off) |  |
| 25 | 1149-W21a-18 | geometric | Backed and oblique fragment | low | sharp lateral | shaving wood | edge removals |  |
| 26 | 1451-V21b-1 | non geometric | Retouched/backed fragment | low | not diagnostic | not diagnostic | not diagnostic |  |
| 27 | 1150-V21b-1 | geometric | Asymmetrical trapeze A | low | distal end | projectile tip | DIF (bending step) |  |
| 28 | 1150-V21b-2 | geometric | Backed and oblique fragment | low | not diagnostic | not diagnostic | not diagnostic |  |
| 29 | 1150-V21b-3 | non geometric | Obliquely truncated bladelet | low | distal end | projectile tip | DIF |  |
| 30 | 1150-V21b-4 | geometric | Backed and oblique fragment | low | sharp lateral | shaving wood | Edge removals | Bright spot |
| 31 | 1150-V21b-5 | geometric | Backed and oblique fragment | PDSM | PDSM | PDSM | PDSM |  |
| 32 | 1150-V21b-6 | non geometric | truncated fragment |  | not diagnostic | not diagnostic | not diagnostic |  |
| 33 | 1466-V21b-1 | non geometric | Alternately retouched bladelet | low | distal end | projectile tip | DIF (bending positive) | Bright spot |
| 34 | 1466-V21b-2 | geometric | Backed and straight fragment | low | not diagnostic | not diagnostic | not diagnostic |  |
| 35 | 1466-V21b-3 | non geometric | Retouched/backed fragment | low | distal end | projectile tip | DIF (bending hinge) |  |
| 36 | 1466-V21b-4 | geometric | Backed and straight fragment | high | distal end | projectile tip | DIF (bending feather) |  |
| 37 | 1466-V21b-5 | geometric | Backed and oblique fragment | low | proximal end | projectile tip | DIF (bending step) | Bright spot |
| 38 | 1466-V21b-6 | non geometric | Retouched/backed fragment | low | both ends | projectile tip | DIF |  |
| 39 | 1509-V22d-1 | geometric | Trapeze with one straight end | high | no use-wear | no use-wear | no use-wear |  |
| 40 | 1509-V22d-2 | non geometric | Retouched/backed fragment | low | not diagnostic | not diagnostic | not diagnostic |  |
| 41 | 1509-V22d-3 | non geometric | Retouched/backed fragment | high | no traces | no traces | no traces |  |
| 42 | 1509-V22d-4 | geometric | Backed and straight fragment | low | sharp lateral | projectile barb?? | DIF (bending step) |  |
| 43 | 1509-V22d-5 | non geometric | Retouched/backed fragment | low | not diagnostic | not diagnostic | not diagnostic |  |
| 44 | 1509-V22d-6 | non geometric | Retouched/backed fragment | low | not diagnostic | not diagnostic | not diagnostic |  |
| 45 | 1509-V22d-7 | geometric | Backed and straight fragment | low | not diagnostic | not diagnostic | not diagnostic |  |
| 46 | 1509-V22d-8 | geometric | Backed and straight fragment | low | proximal end | projectile tip | DIF (burination) |  |
| 47 | 1509-V22d-9 | non geometric | Backed and oblique fragment | high | sharp lateral | cutting and scraping | edge removals |  |
| 48 | 1509-V22d-10 | non geometric | Retouched/backed fragment | low | not diagnostic | not diagnostic | not diagnostic |  |
| 49 | 1509-V22d-11 | non geometric | Retouched/backed fragment | high | sharp lateral | shaving wood | edge removals and polish |  |
| 50 | 1509-V22d-12 | non geometric | Retouched/backed fragment | low | not diagnostic | not diagnostic | not diagnostic |  |
| 51 | 1458-V22d-1 | non geometric | Retouched/backed fragment | high | proximal lateral | projectile tip | DIF (crushing) |  |
| 52 | 1458-V22d-2 | non geometric | Alternately retouched bladelet | low | not diagnostic | not diagnostic | not diagnostic | Edge removals |
| 53 | 1458-V22d-3 | non geometric | Retouched/backed fragment | high | no traces | no traces | no traces |  |
| 54 | 1152-V22d-1 | geometric | Backed and oblique fragment | low | distal end | projectile tip | DIF (bending hinge) |  |
| 55 | 1152-V22d-2 | non geometric | Retouched/backed fragment | low | not diagnostic | not diagnostic | not diagnostic |  |
| 56 | 1152-V22d-3 | geometric | Backed and straight fragment | high | not diagnostic | not diagnostic | not diagnostic |  |
| 57 | 1426-W21a-1 | geometric | Backed and straight fragment | high | sharp lateral | cutting meat | edge removals |  |
| 58 | 1426-W21a-2 | geometric | Backed and oblique fragment |  | sharp lateral | cutting meat | edge removals |  |
| 59 | 1426-W21a-3 | geometric | Backed and straight fragment | low | not diagnostic | not diagnostic | not diagnostic |  |
| 60 | 1426-W21a-4 | non geometric | Retouched/backed fragment | low | distal end | drill/engraver | edge removals |  |
| 61 | 1426-W21a-5 | non geometric | Retouched/backed fragment | high | proximal end | projectile tip | DIF (bending hinge) |  |
| 62 | 1426-W21a-6 | geometric | Backed and oblique fragment | low | distal end | projectile tip | DIF (burination) and striations |  |
| 63 | 1426-W21a-8 | non geometric | Retouched/backed fragment | low | both ends | projectile tip | DIF (bending step) |  |
| 64 | 1426-W21a-9 | geometric | Backed and oblique fragment | low | distal end | projectile tip | DIF (bending step) |  |
| 65 | 1471-W21a-1 | geometric | trapeze | high | PDSM | PDSM | PDSM |  |
| 66 | 1471-W21a-3 | geometric | Straight truncated and backed | low | proximal end | projectile tip | DIF (spin-off) | Edge removals |
| 67 | 1471-W21a-4 | geometric | Backed and oblique fragment | PDSM | PDSM | PDSM | PDSM |  |
| 68 | 1471-W21a-5 | non geometric | Retouched/backed fragment | low | distal end | projectile tip | DIF (bending step) |  |
| 69 | 1471-W21a-6 | geometric | Backed and straight fragment | low | sharp lateral | cutting soft material | edge removals |  |
| 70 | 1471-W21a-7 | non geometric | Retouched/backed fragment | low | not diagnostic | not diagnostic | not diagnostic |  |
| 71 | 1471-W21a-8 | non geometric | Retouched/backed fragment | high | sharp lateral | projectile barb | DIF (crushing) |  |
| 72 | 1471-W21a-9 | non geometric | Retouched/backed fragment | low | both ends | projectile tip | DIF (bending hinge) |  |
| 73 | 1431-W22c-1 | geometric | Backed and oblique fragment | high | sharp lateral | filleting meat | edge removals |  |
| 74 | 1431-W22c-2 | geometric | Backed and oblique fragment | high | distal end | projectile tip | DIF (bending hinge) |  |
| 75 | 1472-W22c-1 | non geometric | pointed backed bladelet | low | not diagnostic | not diagnostic | not diagnostic |  |
| 76 | 1472-W22c-2 | non geometric | Retouched/backed fragment | low | hafted piece | hafted piece | bright spot and edge removals |  |
| 77 | 1472-W22c-3 | geometric | Backed and oblique fragment | low | not diagnostic | not diagnostic | not diagnostic |  |
| 78 | 1472-W22c-4 | geometric | Backed and straight fragment | low | sharp lateral | shaving wood | edge removals and polish |  |
| 79 | 1151-W22c-1 | non geometric | Retouched/backed fragment | low | distal end | projectile tip | DIF (bending step) |  |
| 80 | 1151-W22c-2 | geometric | Backed and straight fragment | low | proximal end | projectile tip | DIF (bending step) |  |
| 81 | 1151-W22c-3 | non geometric | Retouched/backed fragment | low | both ends | projectile tip | DIF (bending step and bending hinge) |  |
| 82 | 1151-W22c-4 | geometric | Backed and straight fragment | low | proximal end | projectile tip | DIF (bending step and spin-off) |  |
